# Accumulated Epinephrine Dose is Associated With Acute Kidney Injury Following Resuscitation in Adult Cardiac Arrest Patients

**DOI:** 10.3389/fphar.2022.806592

**Published:** 2022-01-20

**Authors:** Qiang Gao, Hsiao-Pei Mok, Hai-Long Qiu, Jianzheng Cen, Jimei Chen, Jian Zhuang

**Affiliations:** ^1^ Department of Cardiac Surgery, Guangdong Cardiovascular Institute, Guangdong Provincial People’s Hospital, Guangdong Academy of Medical Sciences, Guangzhou, China; ^2^ Department of Breast Cancer, Guangdong Provincial People’s Hospital, Guangdong Academy of Medical Sciences, Guangzhou, China

**Keywords:** cardiac arrest, cardiopulmonary resuscitation, acute kidney injury, epinephrine, cohort study

## Abstract

The goal of this study was to investigate the association between total epinephrine dosage during resuscitation and acute kidney injury after return of spontaneous circulation in patients with cardiac arrest. We performed a secondary analysis of previously published data on the resuscitation of cardiac arrest patients. Bivariate, multivariate logistic regression, and subgroup analyses were conducted to investigate the association between total epinephrine dosage during resuscitation and acute kidney injury after return of spontaneous circulation. A total of 312 eligible patients were included. The mean age of the patients was 60.8 ± 15.2 years. More than half of the patients were male (73.4%) and had an out-of-hospital cardiac arrest (61.9%). During resuscitation, 125, 81, and 106 patients received ≤2, 3 - 4, and ≥5 mg epinephrine, respectively. After return of spontaneous circulation, there were 165 patients (52.9%) and 147 patients (47.1%) with and without acute kidney injury, respectively. Both bivariate and multivariate analysis showed a statistically significant association between total epinephrine dosage and acute kidney injury. The subgroup analysis showed that the strength of the association between epinephrine dosage and acute kidney injury varied by location of cardiac arrest. Further multivariate regression analysis found that the association between epinephrine dosage and acute kidney injury was only observed in patients with in-hospital cardiac arrest after adjusting for multiple confounding factors. Compared with in-hospital cardiac arrest patients who received ≤2 mg of epinephrine, patients with 3–4 mg of epinephrine or ≥5 mg of epinephrine had adjusted odds ratios of 4.2 (95% confidence interval 1.0–18.4) and 11.3 (95% confidence interval 2.0–63.0), respectively, to develop acute kidney injury. Therefore, we concluded that a higher epinephrine dosage during resuscitation was associated with an increased incidence of acute kidney injury after return of spontaneous circulation in adult patients with in-hospital cardiac arrest.

## Introduction

Cardiac arrest (CA) is defined as the sudden cessation of myocardial contractions and circulation to the cardiac tissue, which is determined by a lack of pulse and loss of consciousness (Cummins et al., 1991). CA is an important public health issue (Stecker et al., 2014). It was estimated that 356,500 people had out-of-hospital cardiac arrest and 209,000 had in-hospital cardiac arrest per year in America (Benjamin et al., 2017). The overall prognosis after CA is poor with an approximate 20% survival rate (Sandroni et al., 2016). Patients who survive CA can suffer from multiorgan failure and increased long-term morbidity (Pekkarinen et al., 2019; Yan et al., 2020). These outcomes can greatly impact an individual patient and generate a societal burden. Thus, it is important to explore the underlying pathophysiological changes during CA in order to improve prognosis in the affected patients.

Previous studies have shown that the pathological process of return to spontaneous circulation (ROSC) after CA involves hypoxic-ischemic encephalopathy, myocardial dysfunction, and ischemia-reperfusion injury (Stub et al., 2011), all of which can cause tissue hypoperfusion and multiple organ damage. Due to its sensitivity to ischemia-reperfusion injury and low perfusion, the kidney is easily damaged during the resuscitation process. Even in CA survivors, the incidence of acute kidney injury (AKI) is reported to be approximately 40.3% (range 32.9–47.8%) (Prasitlumkum et al., 2021). Previous studies have shown that AKI following CA was independently related to poor outcomes, including mortality, neurological dysfunction, and cognitive impairment (Sandroni et al., 2016). Sandroni et al. reported that the mortality of patients with AKI was higher than that of patients without AKI (Sandroni et al., 2016). Worsening neurological function was also found in patients with AKI compared to patients without AKI (Brain Resuscitation Clinical Trial I Study Group, 1986).

Compared with the number of studies focused on mortality and neurological function following CA (Sasson et al., 2010), the number of studies concerning acute kidney injury (AKI) following cardiopulmonary resuscitation (CPR) are relatively small (Patyna et al., 2021; Prasitlumkum et al., 2021). Therefore, there is an urgent need to investigate AKI following CA.

Epinephrine is the most important medication used during CPR. Based on the recommendation from the American Heart Association and European Resuscitation Council guidelines for adult patients with CA, the standard dose of epinephrine administration is 1 mg per 3–5 min (Panchal et al., 2020). Epinephrine can increase aortic blood pressure and coronary flow and perfusion pressure during resuscitation (Paradis et al., 1991). However, an increasing number of studies have shown associations between epinephrine administration and severe neurologic defects (Perkins et al., 2018), a lower rate of ROSC, and a decreased survival chance of hospital discharge (Olasveengen et al., 2012). These poor outcomes might relate to the total epinephrine dosage administered during the resuscitation (Sigal et al., 2019). Since AKI frequently occurs and is associated with other organ injuries after ROSC, there is a need to investigate association between epinephrine administration and AKI following CA. In addition, this association might be confounded by other variables. Therefore, we performed the present study to investigate the independent association between the total epinephrine dosage during resuscitation and AKI following resuscitation.

## Methods

### Study Population

We performed a secondary analysis of a previously published retrospective study that was conducted in the intensive care unit (ICU) at Erasme Hospital, Brussels, Belgium, and was approved by the local Ethical Committee (Comite´ d’Ethique Hospitalo-Facultaire Erasme-ULB). The original data from that study are stored in a public domain (https://datadryad.org/) for free access and analysis (https://doi.org/10.5061/dryad.qv6fp83) (Iesu et al., 2018). The initial study included comatose patients (Glasgow Coma Scale, GCS <9) after in-hospital CA or out-of-hospital CA from January 2007 to December 2015. Patients with incomplete data on liver function or death within 24 h after ICU admission were excluded.

In the present study, we further excluded patients <18 years old, with history of chronic kidney failure, or having missing data on the AKI diagnosis. The definition of AKI was based on the Acute Kidney Injury Network criteria. Briefly, the diagnostic criteria of AKI was defined as an absolute increase of serum creatinine more than or equal to 0.3 mg/dl, a 1.5-fold increase in serum creatinine level from baseline, or a reduction in urine output to less than 0.5 ml/kg per hour for more than 6 hours (Mehta et al., 2007).

### Cardiopulmonary Resuscitation and Post-resuscitation Care

CPR was performed following the standard recommendations (Travers et al., 2010). The post-resuscitation treatments were described previously (Tujjar et al., 2015; Iesu et al., 2018). Briefly, targeted temperature management (TTM, target body temperature 32–34 °C) was applied to all comatose patients. Midazolam and morphine were used to maintain deep sedation. A PiCCO machine (Pulsion, Munich, Germany) was used to monitor the hemodynamic status. *Trans*-oesophageal and/or transthoracic echocardiography were used to evaluate the cardiac function. The mean arterial pressure was maintained at > 65–70 mmHg via volume resuscitation and dobutamine and/or noradrenaline infusion. Intra-aortic balloon counterpulsation (IABP) or extracorporeal membrane oxygenation (ECMO) was applied in patients with severe cardiogenic shock. The normocapnia and SpO_2_ > 94% was supported by mechanical ventilation. Blood glucose was controlled between 110 and 150 mg/dl. Enteral nutrition was started during TTM and adjusted according to the gastric tolerance.

### Data Collection

Data included demographics (age, sex, and weight), comorbidities (chronic heart failure, hypertension, coronary heart disease, diabetes, chronic obstructive pulmonary disease, previous neurological diseases, and liver cirrhosis), CPR information (in-hospital CA or out-of-hospital CA, bystander CPR, time to ROSC, total epinephrine dosage, non-shockable rhythm, witness arrest, cardiac etiology), and lactate and C-reactive protein (CRP) levels at admission.

### Statistical Analysis

Mean ± standard deviation (normal distribution) or median Q1-Q3 (skewed distribution) was used for continuous variables. Numerical values and percentages were used for categorical variables. To determine significant differences among groups with different epinephrine dosages, the Chi-square test was applied for the categorical variables and one-way ANOVA or the Mann-Whitney *U* test was applied for continuous variables when appropriate.

The association between different variables and AKI was first investigated using bivariate analysis. The effect of epinephrine dosage on AKI under other potential confounders was evaluated using multivariate logistic regression analysis. The criteria of selecting confounding variables were as follows: 1) There were 165 patients diagnosed with AKI. We selected less than 16 variables to avoid over-fitting the model. 2) Clinical significance of variables was related to AKI after CA. The clinical significance was determined by searching the literature (Sandroni et al., 2016; Neumayr et al., 2017; Cornell et al., 2018; Mah et al., 2021; Patyna et al., 2021) and our clinical experience. 3) Specific variables were selected as potential confounders if they changed the estimates of epinephrine dosage on AKI by more than 10% or were significantly associated with the AKI.

Interaction and stratified analysis were conducted according to age (<50 years; ≥ 50 years, < 60 years; ≥ 60 years, < 70 years; ≥ 70 years), shock, and out-of-hospital cardiac arrest. The test for linear trend was also performed by entering the median value of each category of selected variables as continuous variables in the models.

All analyses were conducted in statistical software packages R (http://www.R-project.org, The R Foundation) and EmpowerStats (http://www.empowerstats.com, X&Y Solutions, Inc., Boston, MA). A *p*-value < 0.05 in a two-tailed test was considered statistically significant.

## Results

**FIGURE 1:**
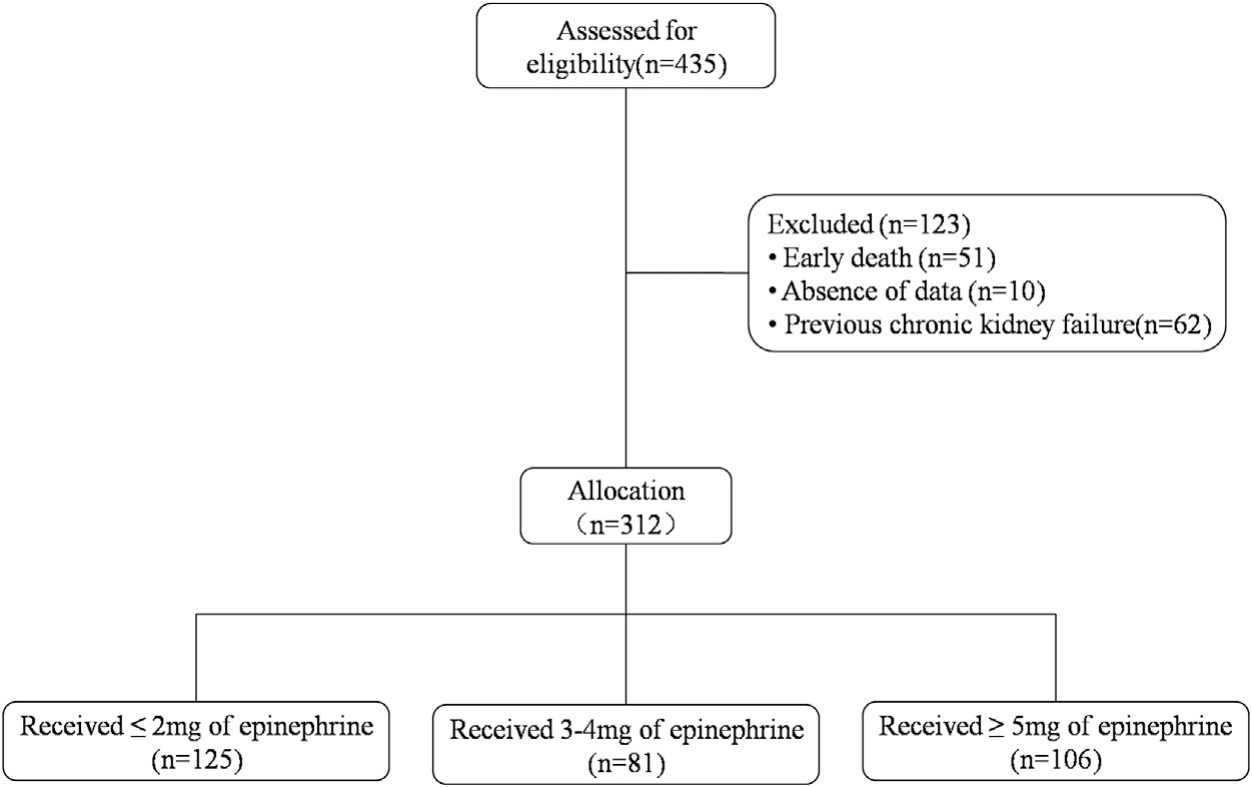
Study participant selection.

A total of 435 patients were screened for inclusion in this study. After excluding 51, 62, and 10 patients due to early death (<24 h), history of chronic kidney failure, and missing data, respectively, 312 patients met the inclusion criteria of the present study (Figure 1). There were 165 patients (52.9%) with AKI and 147 patients (47.1%) without AKI.

### Clinical Characteristics of Study Participants

Table 1 presents the descriptive statistics for the study participants. The mean age of the patients was 60.8 ± 15.2 years. The majority of patients were male (229, 73.4%) and 193 patients (61.9%) had out-of-hospital CA. A total of 165 patients (52.9%) had AKI during the ICU stay.

**TABLE 1:** Characteristics of patients with different epinephrine dosages.

Variables	All participants (*N* = 312)	Epinephrine dosage			*p*
		≤2 mg (N = 125)	3–4 mg (N = 81)	≥5 mg (N = 106)	
Age, year, mean ± standard deviation	60.8 ± 15.2	63.1 ± 15.9	60.8 ± 14.6	58.2 ± 14.5	0.030*
Weight, kg, mean ± standard deviation	77.6 ± 14.4	77.1 ± 14.7	78.4 ± 13.6	77.5 ± 14.7	0.813
ICU length of stay, day, median (IQR)	4.0 (2.0–9.0)	4.0 (2.0–9.0)	4.0 (2.0–8.0)	4.0 (2.0–9.0)	0.867
Time to ROSC, minute, *N* (%)	—	—	—	—	<0.001*
≤20	209 (67.0%)	116 (92.8%)	64 (79.0%)	29 (27.4%)	—
>20	103 (33.0%)	9 (7.2%)	17 (21.0%)	77 (72.6%)	—
Lactate, mEq/L, mean ± standard deviation	6.1 ± 3.2	5.1 ± 2.0	6.1 ± 3.3	7.3 ± 3.8	<0.001*
CRP, mg/dL, median (IQR)	32.0 (10.0–70.0)	39.0 (13.0–89.0)	36.0 (11.0–69.0)	23.5 (10.0–54.5)	0.057
Abnormal baseline creatinine, *N* (%)	118 (37.8%)	40 (32.0%)	32 (39.5%)	46 (43.4%)	0.192
Sex, male, *N* (%)	229 (73.4%)	84 (67.2%)	67 (82.7%)	78 (73.6%)	0.048*
Witnessed, *N* (%)	264 (84.6%)	114 (91.2%)	70 (86.4%)	80 (75.5%)	0.004*
Bystander CPR, *N* (%)	205 (65.7%)	98 (78.4%)	50 (61.7%)	57 (53.8%)	<0.001*
Out-of-hospital, *N* (%)	193 (61.9%)	68 (54.4%)	52 (64.2%)	73 (68.9%)	0.069
TTM, N (%)	280 (89.7%)	105 (84.0%)	77 (95.1%)	98 (92.5%)	0.020*
Cardiac cause, *N* (%)	188 (60.3%)	63 (50.4%)	50 (61.7%)	75 (70.8%)	0.007*
Shockable rhythm, *N* (%)	139 (44.6%)	52 (41.6%)	43 (53.1%)	44 (41.5%)	0.199
ICU mortality, *N* (%)	160 (51.3%)	47 (37.6%)	46 (56.8%)	67 (63.2%)	<0.001*
Hospital mortality, *N* (%)	174 (55.8%)	52 (41.6%)	49 (60.5%)	73 (68.9%)	<0.001*
Favorable neurological outcome at 3 months, *N* (%)	127 (40.7%)	68 (54.4%)	29 (35.8%)	30 (28.3%)	<0.001*
Comorbidities, *N* (%)					
Chronic anticoagulation	53 (17.0%)	24 (19.2%)	16 (19.8%)	13 (12.3%)	0.279
Chronic heart failure	58 (18.6%)	21 (16.8%)	11 (13.6%)	26 (24.5%)	0.130
Hypertension	121 (38.8%)	52 (41.6%)	29 (35.8%)	40 (37.7%)	0.681
Coronary artery disease	125 (40.1%)	41 (32.8%)	39 (48.1%)	45 (42.5%)	0.074
Diabetes	68 (21.8%)	25 (20.0%)	25 (30.9%)	18 (17.0%)	0.061
COPD/asthma	55 (17.6%)	26 (20.8%)	12 (14.8%)	17 (16.0%)	0.474
Neurological disease	45 (14.4%)	25 (20.0%)	11 (13.6%)	9 (8.5%)	0.045*
Liver cirrhosis	13 (4.2%)	4 (3.2%)	3 (3.7%)	6 (5.7%)	0.629
During ICU stay, *N* (%)					
IABP	21 (6.7%)	2 (1.6%)	9 (11.1%)	10 (9.4%)	0.011*
ECMO	36 (11.5%)	3 (2.4%)	11 (13.6%)	22 (20.8%)	<0.001*
Shock	158 (50.6%)	49 (39.2%)	46 (56.8%)	63 (59.4%)	0.004*
Vasopressor therapy	231 (74.0%)	84 (67.2%)	63 (77.8%)	84 (79.2%)	0.077
Inotropic agents	163 (52.2%)	59 (47.2%)	46 (56.8%)	58 (54.7%)	0.332
Mechanical ventilation	308 (98.7%)	122 (97.6%)	80 (98.8%)	106 (100.0%)	0.374
CRRT	37 (11.9%)	11 (8.8%)	9 (11.1%)	17 (16.0%)	0.231
AKI	165 (52.9%)	52 (41.6%)	47 (58.0%)	66 (62.3%)	0.004*

AKI, acute kidney injury; COPD, chronic obstructive pulmonary disease; CRP, C-reactive protein; CRRT, continuous renal replacement therapy; ECMO, extracorporeal membrane oxygenation; IABP, Intra-aortic balloon pump; ICU, intensive care unit; IQR, interquartile range; ROSC, return of spontaneous circulation; TTM, targeted temperature management. *p < 0.05.

### Bivariate Analysis

The results of bivariate analysis are presented in Table 2. Compared with patients without AKI, patients with AKI were more likely to have longer ROSC time, higher epinephrine dosage, elevated lactate and CRP levels, and more frequent use of ECMO, shock, vasopressor, and inotropic agents. AKI patients were also more likely to have abnormal baseline creatinine. In addition, AKI patients had a higher ICU mortality and a higher hospital mortality, with poorer neurological outcomes compared to non-AKI patients.

**TABLE 2:** Bivariate analysis of associations between different variables and AKI.

Variables	Without AKI (*N* = 147)	With AKI (*N* = 165)	Odds ratio (95% CI), *p*
Age, year, mean ± standard deviation	60.0 ± 15.3	61.6 ± 15.1	1.01 (0.99, 1.02) 0.361
Weight, kg, mean ± standard deviation	76.3 ± 15.4	78.7 ± 13.4	1.01 (1.00, 1.03) 0.134
Sex, male, *N* (%)	102 (69.4%)	127 (77.0%)	1.47 (0.89, 2.44) 0.131
ICU length of stay, day, median (IQR)	4.0 (3.0–8.0)	4.0 (2.0–9.0)	1.02 (0.99, 1.04) 0.270
Witnessed, *N* (%)	120 (81.6%)	144 (87.3%)	1.54 (0.83, 2.87) 0.170
Bystander CPR, *N* (%)	95 (64.6%)	110 (66.7%)	1.09 (0.69, 1.75) 0.704
Time to ROSC(>20min), *N* (%)	41 (27.9%)	62 (37.6%)	1.56 (0.96, 2.51) 0.070*
Epinephrine dosage, mg, median (IQR)	3.0 (1.0–5.0)	4.0 (2.0–6.0)	1.10 (1.03, 1.18) 0.006*
Out-of-hospital, *N* (%)	97 (66.0%)	96 (58.2%)	0.72 (0.45, 1.14) 0.157
TTM, N (%)	135 (91.8%)	145 (87.9%)	0.64 (0.30, 1.37) 0.253
Cardiac cause, *N* (%)	81 (55.1%)	107 (64.8%)	1.50 (0.95, 2.37) 0.079
Shockable rhythm, *N* (%)	69 (46.9%)	70 (42.4%)	0.83 (0.53, 1.30) 0.423
ICU mortality, *N* (%)	60 (40.8%)	100 (60.6%)	2.23 (1.42, 3.51) <0.0001*
Hospital mortality, *N* (%)	65 (44.2%)	109 (66.1%)	2.46 (1.55, 3.88), <0.0001*
Favorable neurological outcome at 3 months, *N* (%)	75 (51.0%)	52 (31.5%)	0.44 (0.28, 0.70), <0.0001*
Comorbidities, *N* (%)			
Chronic anticoagulation	22 (15.0%)	31 (18.8%)	1.31 (0.72, 2.39) 0.370
Chronic heart failure	23 (15.6%)	35 (21.2%)	1.45 (0.81, 2.59) 0.208
Coronary artery disease	51 (34.7%)	74 (44.8%)	1.53 (0.97, 2.42) 0.068
Hypertension	55 (37.4%)	66 (40.0%)	1.12 (0.71, 1.76) 0.640
Diabetes	31 (21.1%)	37 (22.4%)	1.08 (0.63, 1.85) 0.775
COPD/asthma	28 (19.0%)	27 (16.4%)	0.83 (0.46, 1.49) 0.534
Neurological disease	24 (16.3%)	21 (12.7%)	0.75 (0.40, 1.41) 0.367
Liver cirrhosis	5 (3.4%)	8 (4.8%)	1.45 (0.46, 4.53) 0.525
During ICU stay, *N* (%)			
IABP	7 (4.8%)	14 (8.5%)	1.85 (0.73, 4.73) 0.196
ECMO	9 (6.1%)	27 (16.4%)	3.00 (1.36, 6.61) 0.006*
Shock	53 (36.1%)	105 (63.6%)	3.10 (1.95, 4.93), <0.0001*
Vasopressor therapy	92 (62.6%)	139 (84.2%)	3.20 (1.87, 5.46), <0.0001*
Inotropic agents	59 (40.1%)	104 (63.0%)	2.54 (1.61, 4.02), <0.0001*
Mechanical ventilation	145 (98.6%)	163 (98.8%)	1.12 (0.16, 8.08) 0.907
Lactate, mEq/L, median (IQR)	4.8 (4.0–6.0)	5.3 (4.1–8.2)	1.15 (1.06, 1.24) 0.001*
CRP, mg/dL, median (IQR)	26.0 (6.0–50.0)	41.0 (18.0–87.8)	1.01 (1.00, 1.01) 0.010*
Presence of an abnormal baseline creatinine, *N* (%)	16 (10.9%)	102 (61.8%)	13.26 (7.23, 24.32), <0.0001*

95% CI, 95% confidence interval; AKI, acute kidney injury; COPD, chronic obstructive pulmonary disease; CRP, C-reactive protein; CRRT, continuous renal replacement therapy; ECMO, extracorporeal membrane oxygenation; IABP, intra-aortic balloon pump; ICU, intensive care unit; IQR, interquartile range; ROSC, return of spontaneous circulation; TTM, targeted temperature management. *p < 0.05.

### Multivariate Logistic Regression Analysis

Multivariate logistic regression was applied to construct three models to investigate the independent effect of epinephrine dosage on AKI after CA. The results are listed in Table 3. In the unadjusted model, compared with ≤2 mg epinephrine, the estimate effect value for 3–4 mg epinephrine was 1.9, suggesting that the likelihood of developing AKI increased by 90% in patients who received 3–4 mg epinephrine compared with patients who received ≤2 mg epinephrine. However, to minimize the confounding effects from other variables, two more models were constructed after adjusting for different confounding factors. In the fully adjusted model (model II), the patients who received >5 mg of epinephrine were 260% more likely to develop AKI than those who received ≤2 mg epinephrine.

**TABLE 3:** Multivariate logistic regression analyses.

Epinephrine dosage	Odds ratio (95% confidence interval), *p*		
	Non-adjusted model	Model I	Model II
≤2 mg	1.0	1.0	1.0
3–4 mg	1.9 (1.1, 3.4) 0.022	1.9 (1.1, 3.4) 0.028	2.0 (1.0, 4.3) 0.062
≥5 mg	2.3 (1.4, 3.9) 0.002	2.4 (1.4, 4.1) 0.001	2.6 (1.1, 6.1) 0.031

Model I adjusted for age and sex. Model II adjusted for age, sex, witnessed arrest, bystander CPR, out-of-hospital, lactate value on admission, CRP value at admission, shock; TTM, vasopressor therapy, inotropic agents, time to ROSC group, and presence of an abnormal baseline creatinine on admission.

### Subgroup Analysis

As shown in Table 4, subgroup analysis showed that the strength of the association between the epinephrine dosage and AKI varied with the location of CA (*p* value for interaction <0.05). For in-hospital CA patients, an increase in the epinephrine dose was significantly correlated with an increased incidence of AKI (*p* value for the trend <0.05).

**TABLE 4:** Subgroup analyses.

Groups	Participant number	Odds ratio (95% confidence interval), *p*			*p* for trend	*p* for interaction
		≤2 mg	3–4 mg	≥5 mg		
Age, years	—	—	—	—	—	0.862
<50	65	1.0	2.4 (0.3, 20.1) 0.411	8.6 (0.4, 209.2) 0.186	0.196	—
≥50, <60	82	1.0	1.8 (0.4, 9.4) 0.477	1.8 (0.3, 12.5) 0.547	0.447	—
≥60, <70	69	1.0	1.2 (0.2, 9.2) 0.834	1.8 (0.3, 10.6) 0.506	0.508	—
≥70	96	1.0	1.7 (0.4, 8.0) 0.505	7.7 (0.7, 82.1) 0.092	0.141	—
Shock	—	—	—	—	—	0.707
No	154	1.0	1.3 (0.4, 4.2) 0.633	3.1 (0.8, 11.8) 0.096	0.144	—
Yes	158	1.0	2.9 (0.9, 8.8) 0.068	3.0 (0.8, 10.7) 0.094	0.051	—
Out-of-hospital	—	—	—	—	—	0.027*
No	119	1.0	4.7 (1.0, 21.8) 0.050	12.9 (2.2, 77.2) 0.005	0.004*	—
Yes	193	1.0	1.3 (0.5, 3.3) 0.642	1.1 (0.3, 3.4) 0.899	0.783	—

Each stratification adjusted for all factors (age, male, witnessed arrest, bystander CPR, vasopressor therapy, inotropic agents, lactate value on admission, CRP value on admission, time to ROSC group, presence of an abnormal baseline creatinine on admission, shock, TTM, and left ventricular assist device) except the stratification factor itself. *p < 0.05.

## Discussion

Epinephrine is the most important medication used during CPR in CA patients. Epinephrine administration during CPR has been recommended by the current American Heart Association and European Resuscitation Council guidelines for adult CA (Travers et al., 2010) and numerous large cohort studies (Perkins et al., 2018). Compared with placebo, epinephrine resulted in a better chance of ROSC, more frequent transport to hospital, a higher rate of ICU treatments, and improved 30-day survival outcomes (Huan et al., 2019). Compared with a standard dosage of epinephrine, a higher dosage might favor ROSC (Vandycke and Martens, 2000) or change to a beneficial rhythm (Woodhouse et al., 1995). However, the side effects of epinephrine, especially a high-dosage epinephrine, have been reported to cause adverse outcomes. For instance, epinephrine could decrease cerebral microcirculation and lung air exchange, as well as increase post-resuscitation myocardial dysfunction (Ristagno et al., 2009). A high epinephrine dosage could also induce coronary artery spasm (Karch, 1989). Epinephrine was also an independent risk factor for unfavorable functional outcomes and in-hospital mortality of CA patients with asystole and pulseless electric activity (Arrich et al., 2012). Tujjar et al. and Domanovits et al. reported that a high epinephrine dosage was an independent risk factor for developing AKI in adult CA survivors (Domanovits et al., 2001; Tujjar et al., 2015). Similar results were also reported in a study that exclusively enrolled infants and children (Neumayr et al., 2017). In the present study, after adjusting for potential confounding variables and stratifying patients by age, shock, and location of cardiac arrest, the multivariate logistic regression demonstrated an association between epinephrine dosage and AKI for patients with in-hospital CA.

Kidney vascular spasms and disrupted intra-kidney hemodynamics (Mauk et al., 1977) or changes in post-resuscitation circulation may underlie the association between epinephrine and AKI might (Chua et al., 2012; Tujjar et al., 2015). However, Chua and others examined the risk associations of AKI after CA in 106 patients aged >16 years. They found that total epinephrine dosage was not independently associated with kidney outcome (Chua et al., 2012). AKI was prone to occur in patients with complex post-resuscitation diseases. They proposed that post-resuscitation cardiogenic shock played a key role in AKI development after CA rather than epinephrine dosage. The chance of developing AKI was rare in patients without post-resuscitation cardiogenic shock. Ischemia-reperfusion injury might not be the main cause of AKI due to the robust capacity of human kidney resistance to warm ischemia and reperfusion injury (Chua et al., 2012). However, in the subgroup analysis of shock, the likelihood of AKI increased with increased epinephrine dosage, as the odds ratio was >1, although this was not statistically significant (*p* values in each group and for the trend and interaction were >0.05). Chua’s study claimed that more than half (51.7%) of the patients with post-resuscitation cardiogenic shock had AKI, while only 6.4% of patients without post-resuscitation cardiogenic shock developed AKI (Chua et al., 2012). However, in the present study, the incidence of patients without post-resuscitation cardiogenic shock who developed AKI was 36.4% (60/165), which was almost 6 times of the 6.4% in Chua’s reports. We believe the pathophysiological process related to AKI development after resuscitation was multi-factorial. In addition to myocardial dysfunction and insufficient tissue perfusion, systemic inflammatory response (Adrie et al., 2002), extensive subcapsular hemorrhages (Hutchens et al., 2010), glomerular barrier dysfunction, and vascular hyperpermeability contributed to AKI (Chang et al., 2013). All of these changes might be directly or indirectly related to epinephrine administration, which was supported by the present study. We minimized the influence from baseline abnormal kidney function since we excluded patients with previous chronic kidney failure in the present study.

The association between epinephrine dose and AKI was varied by CA location (*p* value for interaction >0.05). In-hospital CA patients received more epinephrine, and their chance of developing AKI was higher accordingly (Supplementary Table S1). However, for out-of-hospital CA patients, the trend of getting AKI was disturbed (*p* value for trend >0.05) (Supplementary Table S2). Theoretically, once the CA occurred in the hospital, the rescue treatment was more rapid and effective than in the out-of-hospital setting. There were other factors that might have influenced the rescue outcomes of out-of-hospital CA patients. For example, the exact heart arrest time was unclear. The time for the emergency group arrival varied significantly and the presence of physicians was uncertain (Hatakeyama et al., 2021). Thus, more prospective studies should be designed to clarify the association of AKI and epinephrine dose for out-of-hospital CA patients.

### Strengths and Limitations

We performed a multivariate regression analysis and considered the influences of multiple potential confounders on the relationship between epinephrine use and AKI. However, our study was limited by the observational study design, which was hard to establish a causal relationship between epinephrine dosage and AKI. We performed secondary analysis using a previously collected dataset and were not able to access detailed information, such as the duration of shock and quality or duration of resuscitation, which might be potential confounding factors. The diagnosis of kidney failure was based on the AKIN criteria, but not on the latest Kidney Disease: Improving Global Outcomes (KDIGO) guideline. Further prospective studies should be conducted to verify our results. In addition, although the standard dose of epinephrine administration is 1 mg per 3–5 min, the actual situation might be different due to complexities in the clinical practice, especially under emergent situations. The relationship between epinephrine administration and AKI might vary with each dose and interval during CPR. Unfortunately, the original data did not provide information about these factors. Further studies should focus on the epinephrine dose and interval during resuscitation and investigate their effects on AKI.

In conclusion, after adjusting for multiple confounding factors, a higher epinephrine dosage during resuscitation was associated with an increased incidence of AKI after ROSC in adult patients with in-hospital CA. As epinephrine is an essential medication during CPR, more dedicated studies to optimize epinephrine dosage, frequency, or timing during resuscitation are warranted.

## Data Availability

The original contributions presented in the study are included in the article/Supplementary Material, further inquiries can be directed to the corresponding author.

## References

[B1] Adrie C. Adib-Conquy M. Laurent I. Monchi M. Vinsonneau C. Fitting C. (2002). Successful Cardiopulmonary Resuscitation after Cardiac Arrest as a "Sepsis-like" Syndrome. Circulation 106 (5), 562–568. 10.1161/01.cir.0000023891.80661.ad 12147537

[B2] Arrich J. Sterz F. Herkner H. Testori C. Behringer W. (2012). Total Epinephrine Dose during Asystole and Pulseless Electrical Activity Cardiac Arrests Is Associated with Unfavourable Functional Outcome and Increased In-Hospital Mortality. Resuscitation 83 (3), 333–337. 10.1016/j.resuscitation.2011.10.027 22079948

[B3] Benjamin E. J. Blaha M. J. Chiuve S. E. Cushman M. Das S. R. Deo R. (2017). Heart Disease and Stroke Statistics-2017 Update: A Report from the American Heart Association. Circulation 135 (10), e146–e603. 10.1161/cir.0000000000000485 28122885 PMC5408160

[B4] Brain Resuscitation Clinical Trial I Study Group (1986). Randomized Clinical Study of Thiopental Loading in Comatose Survivors of Cardiac Arrest. N. Engl. J. Med. 314 (7), 397–403. 10.1056/nejm198602133140701 2868412

[B5] Chang C. F. Li C. J. Ko C. J. Teng T. H. Lai S. C. Yang M. C. (2013). The post-resuscitative Urinalysis Associate the Survival of Patients with Non-traumatic Out-Of-Hospital Cardiac Arrest. PLoS One 8 (10), e75172. 10.1371/journal.pone.0075172 24124472 PMC3790766

[B6] Chua H. R. Glassford N. Bellomo R. (2012). Acute Kidney Injury after Cardiac Arrest. Resuscitation 83 (6), 721–727. 10.1016/j.resuscitation.2011.11.030 22155699

[B7] Cornell T. T. Selewski D. T. Alten J. A. Askenazi D. Fitzgerald J. C. Topjian A. (2018). Acute Kidney Injury after Out of Hospital Pediatric Cardiac Arrest. Resuscitation 131, 63–68. 10.1016/j.resuscitation.2018.07.362 30075198 PMC6544025

[B8] Cummins R. O. Chamberlain D. A. Abramson N. S. Allen M. Baskett P. J. Becker L. (1991). Recommended Guidelines for Uniform Reporting of Data from Out-Of-Hospital Cardiac Arrest: the Utstein Style. A Statement for Health Professionals from a Task Force of the American Heart Association, the European Resuscitation Council, the Heart and Stroke Foundation of Canada, and the Australian Resuscitation Council. Circulation 84 (2), 960–975. 10.1161/01.cir.84.2.960 1860248

[B9] Domanovits H. Schillinger M. Müllner M. Thoennissen J. Sterz F. Zeiner A. (2001). Acute Renal Failure after Successful Cardiopulmonary Resuscitation. Intensive Care Med. 27 (7), 1194–1199. 10.1007/s001340101002 11534568

[B10] Hatakeyama T. Kiguchi T. Sera T. Nachi S. Ochiai K. Kitamura T. (2021). Physician's Presence in Pre-hospital Setting Improves One-Month Favorable Neurological Survival after Out-Of-Hospital Cardiac Arrest: A Propensity Score Matching Analysis of the JAAM-OHCA Registry. Resuscitation 167, 38–46. 10.1016/j.resuscitation.2021.08.010 34390825

[B11] Huan L. Qin F. Wu Y. (2019). Effects of Epinephrine for Out-Of-Hospital Cardiac Arrest: A Systematic Review and Meta-Analysis of Randomized Controlled Trials. Medicine (Baltimore) 98 (45), e17502. 10.1097/md.0000000000017502 31702610 PMC6855610

[B12] Hutchens M. P. Nakano T. Kosaka Y. Dunlap J. Zhang W. Herson P. S. (2010). Estrogen Is Renoprotective via a Nonreceptor-dependent Mechanism after Cardiac Arrest *In Vivo* . Anesthesiology 112 (2), 395–405. 10.1097/ALN.0b013e3181c98da9 20068453 PMC2821813

[B13] Iesu E. Franchi F. Zama Cavicchi F. Pozzebon S. Fontana V. Mendoza M. (2018). Acute Liver Dysfunction after Cardiac Arrest. PLoS One 13 (11), e0206655. 10.1371/journal.pone.0206655 30395574 PMC6218055

[B14] Karch S. B. (1989). Coronary Artery Spasm Induced by Intravenous Epinephrine Overdose. Am. J. Emerg. Med. 7 (5), 485–488. 10.1016/0735-6757(89)90250-7 2757714

[B16] Mah K. E. Alten J. A. Cornell T. T. Selewski D. T. Askenazi D. Fitzgerald J. C. (2021). Acute Kidney Injury after In-Hospital Cardiac Arrest. Resuscitation 160, 49–58. 10.1016/j.resuscitation.2020.12.023 33450335 PMC7902429

[B17] Mauk R. H. Patak R. V. Fadem S. Z. Lifschitz M. D. Stein J. H. (1977). Effect of Prostaglandin E Administration in a Nephrotoxic and a Vasoconstrictor Model of Acute Renal Failure. Kidney Int. 12 (2), 122–130. 10.1038/ki.1977.89 916501

[B18] Mehta R. L. Kellum J. A. Shah S. V. Molitoris B. A. Ronco C. Warnock D. G. (2007). Acute Kidney Injury Network: Report of an Initiative to Improve Outcomes in Acute Kidney Injury. Crit. Care 11 (2), R31. 10.1186/cc5713 17331245 PMC2206446

[B19] Neumayr T. M. Gill J. Fitzgerald J. C. Gazit A. Z. Pineda J. A. Berg R. A. (2017). Identifying Risk for Acute Kidney Injury in Infants and Children Following Cardiac Arrest. Pediatr. Crit. Care Med. 18 (10), e446–e54. 10.1097/pcc.0000000000001280 28737594 PMC5628129

[B20] Olasveengen T. M. Wik L. Sunde K. Steen P. A. (2012). Outcome when Adrenaline (Epinephrine) Was Actually Given vs. Not Given - Post Hoc Analysis of a Randomized Clinical Trial. Resuscitation 83 (3), 327–332. 10.1016/j.resuscitation.2011.11.011 22115931

[B21] Panchal A. R. Bartos J. A. Cabañas J. G. Donnino M. W. Drennan I. R. Hirsch K. G. (2020). Part 3: Adult Basic and Advanced Life Support: 2020 American Heart Association Guidelines for Cardiopulmonary Resuscitation and Emergency Cardiovascular Care. Circulation 142, S366. 10.1161/CIR.0000000000000916 33081529

[B23] Paradis N. A. Martin G. B. Rosenberg J. Rivers E. P. Goetting M. G. Appleton T. J. (1991). The Effect of Standard- and High-Dose Epinephrine on Coronary Perfusion Pressure during Prolonged Cardiopulmonary Resuscitation. JAMA 265 (9), 1139–1144. 10.1001/jama.1991.03460090087038 1996000

[B24] Patyna S. Riekert K. Buettner S. Wagner A. Volk J. Weiler H. (2021). Acute Kidney Injury after In-Hospital Cardiac Arrest in a Predominant Internal Medicine and Cardiology Patient Population: Incidence, Risk Factors, and Impact on Survival. Ren. Fail. 43 (1), 1163–1169. 10.1080/0886022x.2021.1956538 34315321 PMC8330738

[B25] Pekkarinen P. T. Bäcklund M. Efendijev I. Raj R. Folger D. Litonius E. (2019). Association of Extracerebral Organ Failure with 1-year Survival and Healthcare-Associated Costs after Cardiac Arrest: an Observational Database Study. Crit. Care 23 (1), 67. 10.1186/s13054-019-2359-z 30819234 PMC6396453

[B26] Perkins G. D. Ji C. Deakin C. D. Quinn T. Nolan J. P. Scomparin C. (2018). A Randomized Trial of Epinephrine in Out-Of-Hospital Cardiac Arrest. N. Engl. J. Med. 379 (8), 711–721. 10.1056/NEJMoa1806842 30021076

[B27] Prasitlumkum N. Cheungpasitporn W. Sato R. Chokesuwattanaskul R. Thongprayoon C. Patlolla S. H. (2021). Acute Kidney Injury and Cardiac Arrest in the Modern Era: an Updated Systematic Review and Meta-Analysis. Hosp. Pract. 49 (4), 280–291. 10.1080/21548331.2021.1931234 33993820

[B28] Ristagno G. Tang W. Huang L. Fymat A. Chang Y. T. Sun S. (2009). Epinephrine Reduces Cerebral Perfusion during Cardiopulmonary Resuscitation. Crit. Care Med. 37 (4), 1408–1415. 10.1097/CCM.0b013e31819cedc9 19242339

[B29] Sandroni C. Dell'anna A. M. Tujjar O. Geri G. Cariou A. Taccone F. S. (2016). Acute Kidney Injury after Cardiac Arrest: a Systematic Review and Meta-Analysis of Clinical Studies. Minerva Anestesiol 82 (9), 989–999. 26957119

[B30] Sasson C. Rogers M. A. Dahl J. Kellermann A. L. (2010). Predictors of Survival from Out-Of-Hospital Cardiac Arrest: a Systematic Review and Meta-Analysis. Circ. Cardiovasc. Qual. Outcomes 3 (1), 63–81. 10.1161/circoutcomes.109.889576 20123673

[B31] Sigal A. P. Sandel K. M. Buckler D. G. Wasser T. Abella B. S. (2019). Impact of Adrenaline Dose and Timing on Out-Of-Hospital Cardiac Arrest Survival and Neurological Outcomes. Resuscitation 139, 182–188. 10.1016/j.resuscitation.2019.04.018 30991079

[B33] Stecker E. Reinier K. Marijon E. Narayanan K. Teodorescu C. Uy-Evanado A. (2014). Public Health burden of Sudden Cardiac Death in the United States. Circ. Arrhythm Electrophysiol. 7, 212. 10.1161/CIRCEP.113.001034 24610738 PMC4041478

[B34] Stub D. Bernard S. Duffy S. J. Kaye D. M. (2011). Post Cardiac Arrest Syndrome: a Review of Therapeutic Strategies. Circulation 123 (13), 1428–1435. 10.1161/circulationaha.110.988725 21464058

[B35] Travers A. H. Rea T. D. Bobrow B. J. Edelson D. P. Berg R. A. Sayre M. R. (2010). Part 4: CPR Overview: 2010 American Heart Association Guidelines for Cardiopulmonary Resuscitation and Emergency Cardiovascular Care. Circulation 122, S676–S684. 10.1161/circulationaha.110.970913 20956220

[B36] Tujjar O. Mineo G. Dell'Anna A. Poyatos-Robles B. Donadello K. Scolletta S. (2015). Acute Kidney Injury after Cardiac Arrest. Crit. Care 19 (1), 169. 10.1186/s13054-015-0900-2 25887258 PMC4416259

[B37] Vandycke C. Martens P. (2000). High Dose versus Standard Dose Epinephrine in Cardiac Arrest - a Meta-Analysis. Resuscitation 45 (3), 161–166. 10.1016/s0300-9572(00)00188-x 10959014

[B38] Woodhouse S. P. Cox S. Boyd P. Case C. Weber M. (1995). High Dose and Standard Dose Adrenaline Do Not Alter Survival, Compared with Placebo, in Cardiac Arrest. Resuscitation 30 (3), 243–249. 10.1016/0300-9572(95)00890-x 8867714

[B39] Yan S. Gan Y. Jiang N. Wang R. Chen Y. Luo Z. (2020). The Global Survival Rate Among Adult Out-Of-Hospital Cardiac Arrest Patients Who Received Cardiopulmonary Resuscitation: a Systematic Review and Meta-Analysis. Crit. Care 24 (1), 61. 10.1186/s13054-020-2773-2 32087741 PMC7036236

