# Supplementary material for: Accumulated Epinephrine Dose is Associated With Acute Kidney Injury Following Resuscitation in Adult Cardiac Arrest Patients
Source: Front Pharmacol. 2022 Jan 20;13:806592. doi: 10.3389/fphar.2022.806592 (PMC8811500; doi:10.3389/fphar.2022.806592)
Supplement: Supplementary file 1 [file DataSheet1.doc]

**Supplementary Table 1.** Multivariate logistic regression analyses for patients with in-hospital cardiac arrest

| Epinephrine dosage | Odds Ratio (95% Confidence Interval), P | | |
| --- | --- | --- | --- |
|  | Non-adjusted model | Model I | Model II |
| ≤ 2 mg | 1.0 | 1.0 | 1.0 |
| 3-4 mg | 3.1 (1.2, 7.9) 0.021 | 2.7 (1.0, 7.3) 0.045 | 4.2 (1.0, 18.4) 0.056 |
| ≥ 5 mg | 4.3 (1.7, 11.2) 0.003 | 3.8 (1.4, 10.1) 0.009 | 11.3 (2.0, 63.0) 0.006 |

Model I adjusted for age and sex.

Model II adjusted for age, sex, witnessed arrest, bystander CPR, chronic kidney failure, lactate value on admission, CRP value at admission, shock, TTM, vasopressor therapy, inotropic agents, time to ROSC group, and presence of an abnormal baseline creatinine on admission.

**Supplementary Table 2.** Multivariate logistic regression analyses for patients with out-of-hospital cardiac arrest

| Epinephrine dosage | Odds Ratio (95% Confidence Interval), P | | |
| --- | --- | --- | --- |
|  | Non-adjusted model | Model I | Model II |
| ≤ 2 mg | 1.0 | 1.0 | 1.0 |
| 3-4 mg | 1.5 (0.7, 3.2) 0.243 | 1.6 (0.8, 3.3) 0.217 | 1.4 (0.5, 3.5) 0.532 |
| ≥ 5 mg | 1.8 (0.9, 3.6) 0.076 | 2.0 (1.0, 3.9) 0.053 | 1.2 (0.4, 3.6) 0.802 |

Model I adjusted for age and sex.

Model II adjusted for age, sex, witnessed arrest, bystander CPR, chronic kidney failure, lactate value on admission, CRP value at admission, shock, TTM, vasopressor therapy, inotropic agents, time to ROSC group, and presence of an abnormal baseline creatinine on admission.
